# Potential Applicability of Cocoa Pulp (*Theobroma cacao* L) as an Adjunct for Beer Production

**DOI:** 10.1155/2020/3192585

**Published:** 2020-09-02

**Authors:** Cassiane S. O. Nunes, Marília L. C. da Silva, Geany P. Camilloto, Bruna A. S. Machado, Katharine V. S. Hodel, Maria Gabriela B. Koblitz, Giovani B. M. Carvalho, Ana Paula T. Uetanabaro

**Affiliations:** ^1^Department of Biology and Biotechnology of Microorganisms, State University of Santa Cruz, Ilhéus 45662.900, Brazil; ^2^Bahian Federal Institute Catu Campus, Catu 48110-000, Brazil; ^3^Department of Biotechnology, State University of Feira de Santana, Feira de Santana 44036-900, Brazil; ^4^University Center SENAI CIMATEC, SENAI Institute of Innovation (ISI) in Advanced Health Systems (CIMATEC ISI SAS), National Service of Industrial Learning–SENAI, Salvador 41650-010, Bahia, Brazil; ^5^Food and Nutrition Center, Federal University of the State of Rio de Janeiro, Rio de Janeiro 21941-901, Brazil

## Abstract

The aim of this study was to evaluate the application of cocoa pulp as an adjunct for malt in beer production. The cocoa pulp was analyzed for humidity, proteins, lipids, sugars, total soluble solids, organic acids, and minerals. A study was carried out to reduce the cocoa pulp viscosity by enzymatic depectinization, making its use viable in beer production. The cocoa pulp showed relevant quantities of compounds important in fermentation, such as sugars, acids, and minerals. In fermentation using the adjunct, the proportions of pulp used were 10, 30, and 49%. A significant difference was found between the adjunct and all-malt worts. The 30% cocoa pulp concentration as an adjunct for malt in the fermentation medium contributed the most to the fermentative performance of the yeasts at both 15 and 22°C based on the consumption of apparent extract (°Plato), ethanol production, and cellular growth.

## 1. Introduction

Cocoa (*Theobroma cacao* L.) is known worldwide for the use of its beans in chocolate production [1–4] and is an important commodity for some countries [5–8]. Brazil is a country with a large production of fruits and a high volume of exports, and in the global production of cocoa, Brazil occupied the 6th ranked position in the 2014/2015 harvest [9, 10]. For a long period, the production and commercialization of cocoa were the basis of the economy in some Brazilian states, especially Bahia. Brazil was the second largest producer of cocoa in the world until the appearance of “witch's broom,” a disease caused by the fungus *Moniliophthora perniciosa*, after which the agroindustry of cocoa declined quickly, causing a decrease in the production and exportation of cocoa beans [11–13]. Cocoa pulp is a rich substrate, which can be used in industrial processes to produce by-products [10, 14, 15]. The pulp is made up of a group of mucilaginous spongy cells containing water, fructose, glucose, sucrose, citric acid, and several inorganic salts [16].

Cocoa pulp shows a complex and variable microbiota, and spontaneous fermentation is a prevalent process in the food industry [14, 16–18]. In studies performed with cocoa pulp, it was observed that it can be used as a substrate for the development of different microorganisms, with a higher prevalence of fungus than bacteria, and that within this diversity, there is a trend for *Saccharomyces cerevisiae* to stand out [17]. The use of cocoa as an industrial raw material is advantageous compared to other potentially suitable tropical fruits, as it is an abundant product derived from an established culture. Research aims at obtaining pulp to produce juice, jam, compotes, fermented beverages, and other processed products, thereby offering special and attractive flavors [19].

Cocoa pulp can be easily fermented by yeasts such as *S. cerevisiae* to produce an alcoholic beverage. This yeast has been used in fermentation processes for a long time, according to the first historical reports of beer and wine production [20–27]. The wort composition is very important for beer production because it ensures the quality of the final product and is a growth medium for the yeasts. Several environmental factors influence the production of metabolites and the survival of the yeasts during industrial fermentation. The main factors are temperature, pH, and sugar concentration as well as pulp characteristics in the case of beer production with the use of the adjunct [28–31]. Similar to cocoa pulp, many other fruits can be used in the production of alcoholic beverages [32]. Various experiments have been carried out with fruits such as mango [33], pineapple [34], star fruit [35], banana [36–38], orange [39–41], grape [42], apple [43, 44], cocoa [19], gabiroba [45], kiwi [46], and caja [47] for the production of fermented beverages. However, due to the different chemical compositions of the fruits, further studies are necessary, including the type of fermented beverage to be produced, the ideal temperature of fermentation, and the type of treatment that must be applied to the fruit pulp or the wort in the prefermentative phase and/or during fermentation. Cocoa pulp constitutes a very favorable raw material for the production of beverages by alcoholic fermentation because it is rich in carbohydrates, has low acidity, and contains important organic acids [19, 48–51].

A relevant aspect in the current beer market is the use of adjuncts with a high concentration of fermentable sugars that contribute to higher production of ethanol from each °Plato of extract [52–55]. The use of certain types of adjuncts only contributes to the supply of carbohydrates and no other nutrients; consequently, its addition to the wort at the beginning of beer fermentation results in a similar balance of nutrients to the fermentations without the addition of adjunct [56–58]. The main objective of this work was to evaluate the performance of fermentation in the production of beer using cocoa pulp from the south region of Bahia (Brazil) as an adjunct for malt after the determination of its chemical composition.

## 2. Materials and Methods

### 2.1. Obtaining and Storing the Cocoa Pulp

Cocoa fruits were obtained from clone VP 1151, which was supplied by the Mars Center for Cocoa Science located in the south region of the state of Bahia (Ilhéus, Brazil); the cocoa fruits were harvested at their optimum ripeness for consumption. The selection step consisted of the rejection of defective fruits to avoid compromising the quality of the final pulp and size- and color-based selection from the commercially available fruits. The selected fruits were washed with clean tap water to remove impurities, sanitized for 30 min in a 100 mg·mL^−1^ free chlorine solution, and rinsed with clean tap water to remove excess free chlorine. Then, the cocoa fruits were broken, pulped using a bench depulper (model DM-Ji-05, Macanuda), fractionated, and stored in flexible polyethylene bags. The pulp fractions were then frozen and stored at −18°C until chemical analysis.

### 2.2. Wort Production

The wort was produced according to the conventional techniques of beer production at the Laboratory of Fermentation of the State University of Feira de Santana (UEFS), Bahia, Brazil. During this process, 8.8 kg of malt (Chateau Pilsen 3.25 EBC, from Belgium) was ground in a bench grinder to decrease the grain size and facilitate hydrolysis catalyzed by enzymes during wort production. The wort production ramp used was adapted from Carvalho and Zambiazi [59]. The initial pH value of the wort was adjusted to 5.3 by adding food-grade lactic acid. The wort was hot-packed (>90°C) and stored at 0°C until use.

### 2.3. Chemical Analysis of the Pulp and Wort

The chemical analysis of the cocoa pulp and wort was performed at the Laboratory of Physical-Chemical Analysis of the Department of Technology, State University of Feira de Santana, Bahia. The humidity was determined by direct drying in an oven at 105°C, and ashes were obtained by incinerating the samples in a muffle oven at 550°C. Protein was determined by the Kjeldahl method and lipids by gravimetry after extraction with petroleum ether using a Soxhlet extractor and starch. Pectin and titratable acidity were determined according to the methodology recommended by Adolfo Lutz Institute [60]. The sugars were determined by spectrophotometry, with the reducing sugars established by the DNS method (3,5-dinitrosalicylic acid) [61] and the total sugars by the phenol-sulfuric method [62]. The pH was determined by the potentiometric method using a digital Instrutherm pH meter (model PH-1700) and titratable acidity by neutralization, with the results of the titration expressed in g of malic acid per 100 g of pulp according to the methodology described by Carvalho et al. [63].

The total phenolic content of the cocoa pulp was determined according to Singleton and Rossi [64] by the reaction between the pulp extract and Folin–Ciocalteu reagent in the presence of sodic carbonate, based on the appearance of blue due to the oxidation of phenols in the basic medium. The absorbance was measured in a spectrophotometer (Shimadzu model UV mini 1240) at 765 nm. The quantity of total phenols was expressed in gallic acid equivalents (GAE) (mg GAE/g of sample) through a calibration curve [65]. The color of the wort was determined by the spectrophotometric method at a wavelength of 430 nm [66].

### 2.4. Determination of Sugars and Organic Acids

The sugars and organic acids were determined by high-performance liquid chromatography (HPLC). The samples were diluted 10 times in ultrapure water and filtered through difluoride polyvinylidene membranes (0.45 *μ*m pores, 13 mm diameter) (Millex HV, Millipore). Standard curves were built for the metabolites (glucose, fructose, maltose, maltotriose, citric acid, malic acid, acetic acid, succinic acid, and formic acid) (Sigma-Aldrich), which were diluted in ultrapure water (Direct Q3UV, Millipore, Massachusetts, USA). During the analysis, 5 *μ*L of the sample was used. An UltiMate 3000 (Dionex, Germany) system that was equipped with a UV-Vis detector (at a 210 nm wavelength) for the detection of organic acids and with a refraction index detector for the detection of sugars (Shodex RI-101, Showa Denko, Japan) was used for this analysis. A Rezex ROA Organic Acids ionic exchange column (300 × 7.8 mm) (Phenomenex, Torrance, CA, USA) was used to separate the compounds at a steady temperature of 60°C, and 0.005 M sulfuric acid was used as a mobile phase at a flow rate of 0.6 mL/min. The chromatograms were integrated using Chromeleon Server monitoring software (Dionex). Identification was performed by comparing the retention times, and the quantification was established according to the standard curve for each analyte.

### 2.5. Determination of Minerals

#### 2.5.1. Sample Digestion

For digestion, 0.5 g of the samples was weighed accurately in a glass digestion tube, and 2 mL of concentrated nitric acid was added. The digestion occurred at 120°C for a period of 4 h, and then, 1.5 mL of hydrogen peroxide was added. The resulting solution was quantitatively transferred to a plastic flask and filled to 10 mL with ultrapure water. All samples were analyzed in triplicate to eliminate any specific error, verify the homogeneity of samples, and evaluate the repeatability of the procedure. The blanks were prepared in the same way; however, the sample was omitted for each batch of samples, and the certified reference material was digested using the same method [67].

#### 2.5.2. Inductively Coupled Plasma Optical Emission Spectroscopy (ICP-OES) for Mineral Determination

The ICP-OES operating conditions (Varian, Mulgrave, Australia) were as follows: 15 L/min, argon plasma gas flow rate; 1.5 L/min, auxiliary gas flow rate; 0.7 L/min, nebulizer gas flow rate; 1.3 kW, power; 40 MHz, radio frequency (RF) power; 0.7 mL/min, sample flow rate; and charge-coupled device (CCD), the detector.

All elements were detected in axial mode, and the time of signal integration was 2 seconds. All analytes were measured at two different emission lines. To check for matrix effects on the sensitivity and selectivity, a scan of the emission lines was obtained for both a standard and a digested sample. The emission line was selected, taking into account nonspectral interferences and the best signal to background ratio for all elements, and the background was corrected manually for all emission lines selected. The calibration standards were prepared in a 1.5 mol/L nitric acid solution. The calibration range for all elements was evaluated from 0.02 to 10 m/L, except for calcium, magnesium, potassium, sodium, and phosphorus, for which calibration curves were prepared from 1 to 100 mg/L. The average value for blank samples was subtracted from the analytical signals of digested samples after interpolation on the corresponding calibration graphs.

### 2.6. Viscosity of the Cocoa Pulp: Enzymatic Hydrolysis of the Pectin

In addition to the abovementioned analyses, it is important to highlight that the high viscosity of the cocoa pulp necessitated a study to reduce the viscosity to make its use in the production of beer possible.

The viscosity reduction of the cocoa pulp was performed using polygalacturonase (7900 PGNU/mL) obtained from *Aspergillus niger* and *Aspergillus aculeatus* (Novozymes Pectinex Ultra Clear). A rotational central compound experiment was designed (DCCR) using three variables (enzyme concentration, time, and temperature) and evaluated at two levels using the Statistica 7.0 program.

The maximum temperature was fixed at 80°C, as higher temperatures for a longer period would cause significant water loss, thereby concentrating the pulp and leading to an apparent increase in viscosity compared to the initial pulp. The response variable studied was the percentage reduction of pulp viscosity after treatment with the enzyme.

The experiments consisted of adding the enzyme to 100 g of pure pulp and heating in a static water bath for a preestablished time at a preestablished temperature. After the reaction time, the samples were cooled to 30°C and examined for viscosity in a viscometer (Rheometer Brookfield DVII + Pro). These values were used to calculate the reduction of viscosity using the following equation:

(1) $$\begin{array}{c} \%\;\mathrm{reduction}\text{ of viscosity}=\frac{\left(\text{initial viscosity}\right)-\left(\text{final viscosity}\right)}{\left(\text{initial viscosity}\right)}\times 100. \end{array}$$

The results of the viscosity reduction were processed by the Statistica 7.0 program to obtain a variance analysis table, regression coefficients, and response surface graphs, as well as contour curves.

### 2.7. Yeast Strains

The commercial yeast strains used in this work were lager (Safale S-23, Belgium) and ale (Safale S-04, Belgium).

### 2.8. Conditions of Fermentation

To evaluate the effect of the cocoa pulp as an adjunct for beer production, a follow-up of the fermentation was carried out, analyzing the following parameters: substrate consumption, ethanol production, and cellular concentration.

The propagation of yeasts was realized using 12°P all-malt wort. For initial inoculation, the yeasts were weighed according to the manufacturer's instructions, propagated in an Erlenmeyer flask, and incubated in a rotatory shaker (100 mL, 30°C, 150 rpm, 24 h) with enough agitation to provide a cellular concentration of 1.0 × 10^8^ cell/mL. This aimed to ensure the beginning of fermentation with a 1.0 × 10^7^ cell/mL laboratory scale in a 500 mL Erlenmeyer flask at a useful volume of 250 mL.

### 2.9. Beer Production Using Cocoa Pulp as an Adjunct

The assays for beer production at laboratory scale were performed with a combination of three concentrations of cocoa pulp (10, 30, and 49%) as the adjunct and all-malt wort (0%), two commercial yeasts at 15 (Safale S-23, Belgium) and 22°C (Safale S-04, Belgium), and the concentration of the 12°P original wort. The cocoa pulp went through an enzymatic treatment for viscosity reduction before being added to the brewing wort. Then, the cocoa pulp was added to the wort as an adjunct after the boiling phase, at the beginning of fermentation. The fermentations were performed at 15 and 22°C in a 500 mL Erlenmeyer flask using an airlock valve to seal the system. These fermentations were measured at regular 12 h intervals until the apparent attenuation reached approximately 70–75% (1.0°P above the final value of fermentable sugars). Statistical analysis (ANOVA) was performed by the *F*-test, and the averages were compared by Tukey's test with 5% probability using the statistical software SISVAR 5.0.

### 2.10. Wort Analyses

Throughout the fermentation, samples were collected in triplicate. The cells were removed by centrifugation at 4000 rpm for 10 min, and the supernatant was used at 20°C to quantify the apparent extract and the ethanol concentration by using beer analyzer equipment (bench hydrometer, Rudolph Research Analytical, Tecnal).

### 2.11. Yeast Analyses

The cell concentration in suspension was determined using a Neubauer counting chamber and expressed in cell/mL. The cellular viability was determined by a methylene blue staining method [68]. Each determination was performed in triplicate.

## 3. Results and Discussion

### 3.1. Characterization of the Cocoa Pulp

The cocoa pulp was obtained by mechanical processes and chemically characterized to evaluate its contribution to the process of beer fermentation. The pulp presented high humidity and an average pH value of 3.5. The cocoa pulp, due to its low pH, is a product classified as highly acidic (pH < 4.5), as are most tropical fruits [19]. The concentrations of total and reducing sugars were 18% and 10.4%, respectively (Table 1). Based on these results, the cocoa pulp can be compared to other fruits with potential for use in the beverage industry. The concentration of total solids, determined through the °Brix value, was 17 (Table 1), higher than the soluble solids value for traditional beers (12°Brix) [24]. Studies of cocoa pulp performed by Dias et al. [19], Penha and Matta [69], and Puerari et al. [48] found similar values. In this case, it was necessary to dilute the pulp to standardize the process of beer production, so a sugaring process was not needed, which, according to Dias et al. [47], consists of adjusting the soluble solid content by using a sucrose solution. This adjustment is needed for pulps that have a lower content of soluble solids than the wort for the production of beer. The soluble solid content is a parameter used as an indirect measure of the sugar content [63]. Sugars and organic acids (Table 1) are important compounds of the cocoa pulp that are present during fermentation. The glucose and fructose contents were 52.11 and 52.35 g/L, respectively. The fermentable sugars are those that can contribute to the nutritional composition of the wort. The sugars glucose and fructose, which are found in the cocoa pulp, will contribute to the fermentative process once they are assimilated through the yeasts by facilitated diffusion, stopping the transport process when the concentrations are equal inside and outside the cell [24, 25].

*S. cerevisiae* excretes an extracellular enzyme that hydrolyses sucrose into glucose and fructose, which are then conducted inside the cell. These compounds, found in the cocoa pulp, are important sources of carbon for the formation of products and subproducts that influence the aroma, taste, and final characteristics of the beer. The organic acids citric and malic are commonly found in fermented beverages that contain fruits as preservatives, and they possess antimicrobial properties [19–25]. Thus, the presence of cocoa pulp can contribute positively in terms of nutrients to the microorganisms and sensory characteristics, in addition to having an interesting commercial appeal. In this work, citric and malic acids (organic acids) were found in higher quantities, at 8.33 and 6.10 g/L, respectively.

Acetic acid, a product of the yeast's secondary metabolism, is associated with an “off flavor” in beer and was found in lower quantity. This substance is metabolized exclusively by the yeasts, which reabsorb it at the end of fermentation, altering its concentrations and making them variable at the end of fermentation [24, 70]. Other important nutrients in fermentation are the nitrogen compounds [24, 57]. The value of protein found in the cocoa pulp was 0.62%, which can contribute to the amino acids that are essential for yeast nutrition during fermentation, as well as flavor compound formation in the beer. Carvalho et al. [37], who studied the performance of banana in beer production, obtained a pH of 4.4 to 4.6, an acidity of 0.22 to 0.57% for malic acid, a starch content of 0.9 to 7%, and total soluble solids of 28% [71]. Comparing those values with those of cocoa pulp, this fruit shows compatible values that make it viable for beer production.

### 3.2. Mineral Content of the Cocoa Pulp

The results obtained from the analysis of the minerals in cocoa pulp are found in Table 2. The elements Ca, Cr, Cu, Fe, K, Mg, Mn, Mo, Na, P, Se, and Zn were found in higher quantities than the detection and quantification limits (Table 2), except for Al and Cd, which were found in lesser quantities of 1.74 and 0.14 mg/kg, respectively. Co, Li, Pb, and V were not found in the analyzed sample. The elements found in higher quantities were K, Mg, Na, and P. With respect to the elements Ca and Mg, the magnesium content found in cocoa pulp was the same as in studies performed with kaki [67]; however, the calcium level was lower. These mineral elements are important during fermentation and act as essential cofactors for the activity of several enzymes.

Most of the mineral compounds considered important for yeast performance during fermentation were found in the cocoa pulp. According to Briggs et al. [24], the essential minerals include B, Ca, Co, Cu, Fe, K, Mo, Mn, Mg, Ni, and Zn and the concentrations required for the growth of yeasts are normally lower than 10 *μ*M. Among these minerals, only Co was not found in the pulp. The performance of the yeast during fermentation depends on the complex interactions among the elements potassium, magnesium, and calcium. During beer fermentation, Mg has an important role in the maintenance of yeast membrane integrity when the yeasts are exposed to ethanol. The increase in the bioavailability of Mg before or during fermentation increases cell growth and viability. Supplementing the fermentation medium with Mg has caused an increase in the rate of fermentation and productivity of ethanol. Calcium also contributes to cell growth and metabolic responses to external stimulation and is related to flocculation [72]. Copper and iron act as cofactors of several enzymes, including the redox pigments of the respiratory chain. In low concentrations, copper is a necessary trace element that performs an important and positive role for almost all organisms [73, 74]. However, in high concentrations, copper can exert an inhibitory effect and cause toxicity [75]. Studies have demonstrated that when the copper concentration is greater than 20 mg/L, the growth of *S. cerevisiae* is inhibited, which delays the fermentative process and reduces alcohol production.

According to Sun et al. [76], at a concentration of copper between 9.6 and 19.2 mg/L, there was no impact on the growth of *S. cerevisiae* cells in the production of wine, which differs from the results of studies performed with high copper content (32–96 mg/L) [77, 78]. The concentration of copper can reduce the capacity for sugar absorption, ethanol production, and growth of *S. cerevisiae* strains [78]. The composition of the wort influences the performance of yeasts during fermentation. It is important to highlight that other components found in this composition can influence the fermentation performance. The cocoa pulp can contribute to the wort composition by providing fundamental minerals for the metabolism of yeast in the beer fermentation medium, consequently contributing to the fermentative performance.

### 3.3. Viscosity of the Cocoa Pulp

This assay aimed at finding favorable conditions for the hydrolysis of pectin, consequently reducing the viscosity of the cocoa pulp in order to facilitate its use in beer production wort. Examining the rheological characteristics, it was noted that cocoa pulp has a pseudoplastic behavior. Many factors affect the rheological behavior of fruit pulps, notably temperature, soluble solids, and particle size. Studies show that fruit pulps behave as a pseudoplastic fluid as a result of complex interactions among the soluble sugars, pectic substances, and suspended solids [79].

The pulp showed an apparent high viscosity of 95 cP at 30°C. This characteristic indicates the need for an adequate dimensioning of pumps, pipes, and equipment in order to avoid losses, incrustations, and contamination during handling. Carvalho et al. [37] used an exogenous enzymatic complex to reduce the viscosity of banana pulp in beer production and favor its use in the technological process.

The rheological behavior and flux properties of fruit pulp have a significant role in the food industry, regulating the development of the product as well as the design and evaluation of equipment. In addition, knowing the rheological properties, fundamental to any type of food, can be an indication of how the item behaves under several conditions of a process. Below are the data showing the percentage of viscosity reduction based on experimental planning using the enzyme polygalacturonase from *Aspergillus niger* (Table 3).

Tables 1S and 2S (Supplementary Materials) show the results of the variance analysis for the assay and the regression coefficients with all the factors and their interactions, respectively. The percentage of viscosity reduction varied from 0 (assay 12) to 60.76% (assay 14), with high variability among the 18 assays. The value of R2 (92.2%) considering only the significant factors, as shown in Table 3S (Supplementary Materials), and the *F*-test (Table 4S—Supplementary Materials), indicate that the experiment is significant at the 1% significance level and that the model obtained for predicting the viscosity reduction in relation to the significant variables (equation (2)) can be used to represent the behavior of the viscosity reduction of the cocoa pulp when treated with the enzyme polygalacturonase, within the range studied. In equation (2), *Ŷ* is the response of the percentage of reduction in viscosity of the cocoa pulp and *X*2 and *X*3 are the codified factors temperature and time of action of the enzyme on the cocoa pulp, respectively:

(2) $$\begin{array}{c} \hat{Y}=-12.92X2-10.28X22-4.64X2X3+53.57. \end{array}$$

### 3.4. Model Validation

**Figure 1:**
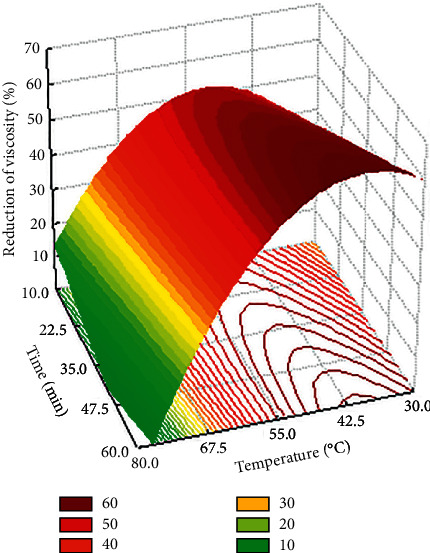
Response surface.

**Figure 2:**
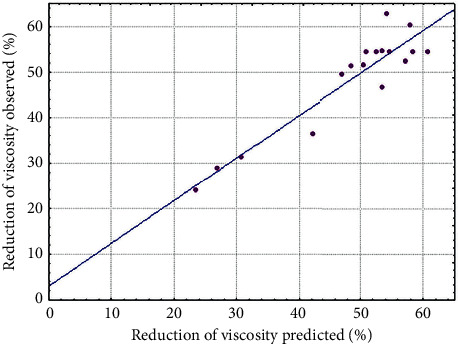
Observed values × predicted values of percentage of reduction in cocoa pulp.

The adequacy of the proposed model (equation (2)) for enzymatic depectinization and, consequently, viscosity reduction, was evaluated using three random conditions located within the optimal region (Figure 1). Hydrolyses were performed in triplicate, with the addition of 500 *μ*L of enzymatic solution for 100 g of pulp. The experimental conditions (Figure 2), the predicted values, and the results of the percentage of reduction in viscosity found are shown in Table 4. Viscosity reductions of 64.92%, 64.75%, and 65.71% were experimentally achieved for assays 1, 2, and 3, respectively, which is in good agreement with the reduction predicted by the model. There was no significant difference among the three assays, which is in accordance with expectations, as the three conditions point to the same percentage of reduction in viscosity, after analysis of Figure 1.

Finally, any condition located within the optimal region can be used to treat cocoa pulp to be incorporated in beer production, as the percentage of reduction in viscosity of approximately 65% was satisfactory; it increased the fluidity of the wort compared to the wort using untreated pulp. In this experiment, it was noted that the increase in temperature provided an increase in the fluidity of the cocoa pulp with the addition of the enzymatic complex, reflecting a reduction in the apparent viscosity. The enzyme acted in the pectic substances, which can form gels in an acidic medium and in the presence of sugar. Due to the presence of pectic substances, the viscosity increases, leading to difficulties in clarification and concentration of juices [80].

In the cocoa pulp used in the present study, 0.51% pectin was found. This finding is in accordance with the value observed by Dias et al. [19], who added an enzymatic complex to reduce viscosity in a study of wine making using cocoa pulp. Manohar et al. [81] studied the properties of mango pulp flow and reported that the pectin content had a marked effect on viscosity; a 5.7% reduction in the content of pectin reduced viscosity by approximately 50%. It is important to note that the quantification of pectin in alcoholic beverages from fruits is important because of the action of pectinesterase enzymes, which release methanol. Methanol inhibits the growth of *S. cerevisiae*, even at low concentrations [4].

### 3.5. Chemical Analysis of the Beer Wort Using Cocoa Pulp

The results of the chemical analysis of the wort and the statistical significances obtained through the *F*-test are shown in Tables 5 and 6, respectively. The mixtures of cocoa pulp and wort (10, 30, and 49%) showed significant differences compared to the wort without the pulp (0%) with respect to the pH, total titratable acid, humidity, soluble solids, protein, and color. The pH of the wort demonstrated the interference of the cocoa pulp with the increase in concentration; therefore, the total acidity was also influenced. It was also noted that the formulation with the higher proportion of pulp (49%) presented a lower pH and higher total acidity. Since the pH values must have an inverse relationship with the total acidity, these results are expected. An explanation for this finding is that the pulp has a high acidity, with the predominance of citric and malic acids (Table 5) that decrease the pH of the mixtures.

ptThe ideal pH to start alcoholic fermentation of beer is from 5.0 to 5.5; therefore, it is necessary to correct the pH of the pulp by adding calcium carbonate to standardize the value. The acidity of the medium influences the activity of the yeasts [24]. Fruit pulps normally show high humidity, and its influence can be observed in the mixture with higher pulp content. The essential nutrients for the fermentation of yeasts are amino acids and fermentable sugars [82]. The protein added to cocoa pulp did not significantly influence the mixtures compared to the 100% all-malt wort. With regard to the concentration of total and reducing sugars, a significant increase in the 30 and 49% cocoa pulp mixtures was observed.

When analyzing the concentration of the fermentable sugars glucose, fructose, maltose, and maltotriose, the influence of the cocoa pulp in relation to glucose and fructose was noted in higher concentrations. The sugars maltose and maltotriose are only derived from the wort and are therefore reduced in the medium of fermentation with the increase in pulp addition, which provides a dilution. During fermentation, the yeasts first consume the hexoses glucose and fructose, followed by the more complex sugars maltose and maltotriose. High concentrations of glucose during fermentation can interfere with the metabolism of maltose, producing a beer with a high concentration of residual sugars in addition to interfering negatively with the fermentative performance [83, 84]. The organic acids are other important elements during fermentation. The contribution of acetic, succinic, and formic acids was not observed in the use of cocoa pulp (Table 6).

An important parameter of beer characterization is the color. According to Briggs et al. [24], the color of beer is related to the melanin and caramel present in the malt. Therefore, the mixtures with a higher concentration of pulp (less malt in the formulation) showed a lower concentration of these pigments and were consequently clearer. It is important to highlight that the cocoa pulp has a clear color, which can also influence the clarification of the mixture.

### 3.6. Analysis of Fermentation of the Beer Wort with Cocoa Pulp as an Adjunct at Concentrations of 10, 30, and 49%

The addition of cocoa pulp as a malt adjunct was used as an alternative method to increase the concentration of soluble solids in the wort, favoring fermentation and beer production with differentiated organoleptic characteristics. The fermentations were performed at concentrations of 10, 30, and 49% cocoa pulp using the commercial yeasts to define the best conditions for the process.

**Figure 3:**
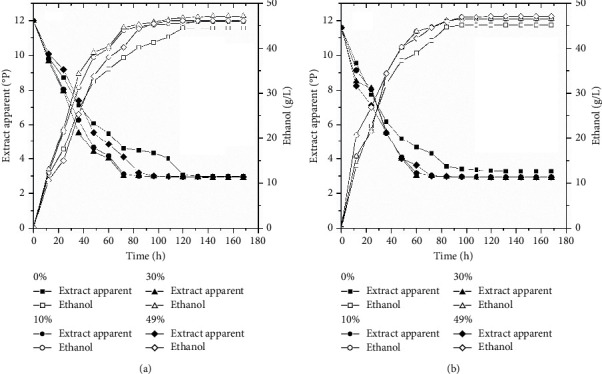
Consumption of apparent extract in all-malt wort 0% and wort with cocoa pulp as an adjunct at concentrations of 10%, 30%, and 49% and ethanol production at concentrations of 0%, 10%, 30%, and 49% of cocoa pulp with the following conditions: initial concentration of wort at 12°P and temperature at 15°C (a) and 22°C (b).

**Figure 4:**
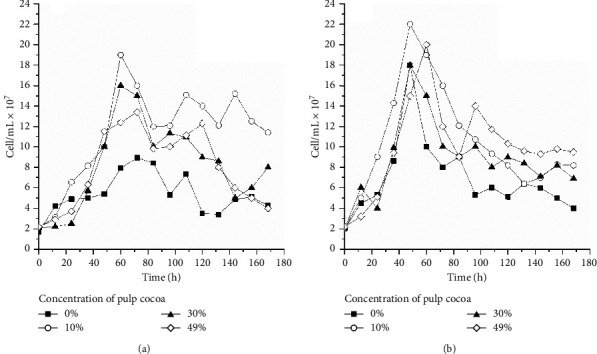
Growth yeasts S-23 and S-04 in all-malt wort 0% and wort with cocoa pulp as an adjunct at the concentrations of 10%, 30%, and 49% in the following conditions: initial concentration of 12°P and temperature at 15°C (a) and 22°C (b).

In the fermentation at laboratory scale using all-malt wort and cocoa pulp as an adjunct, changes in the scale of the wort, production of ethanol, and cells in suspension were evaluated during fermentation at 15 and 22°C, as shown in Figures 3 and 4.

The content of the extract for all fermentations tested was adjusted to 12°P, and the process was ended after the final constant concentration.  Figure 3 reveals the production of ethanol in the first hours of fermentation, with a considerable increase from 48 to 36 h at 15 and 22°C, respectively. The use of the adjunct favored this increase in production. The same behavior was observed in all assays, where the values of ethanol reached 4.5% in the final product when the concentration of the initial apparent extract was 12°P in an all-malt wort [24]. During the first 48 h at 15°C, the consumption of extract (measured in terms of apparent extract) was associated with the increase in the number of cells in suspension (Figures 3 and 4). In this interval (0 to 48 h), the apparent extract decreased approximately 50%, whereas the concentration of cells in suspension increased 20 times. Consequently, the production of ethanol reached 4% (*v*/*v*). At 22°C, the consumption of 50% of the substrate was faster (0 to 36 h), and the cells in suspension increased their growth 22 times, reaching the same volume of ethanol. From 60 h, when the quantity of saturation in oxygen in the medium probably fell, an increase in the production of ethanol was observed for the referred wort. The concentration of ethanol kept increasing in the medium since it is more easily formed under anaerobic conditions. The end of fermentation at 15°C occurred at 120 h, whereas at 22°C, it occurred at 96 h, with a reduction in the apparent extract and concentration of cells and with a subsequent increase in the volume of ethanol.

The performance of beer fermentation during production depends on the capacity of the yeasts to adapt to the changes and conditions imposed by the medium (oxygen availability, osmotic stress, CO_2_ build-up, nutrient limitations, and ethanol toxicity) [82]. An improvement in the performance of the yeasts during fermentation and a reduction in the time of the process are desirable for improving not only productivity but also the efficiency and sustainability of brewing [85]. The analysis of the viable cells as well as of their performance during fermentation is important to ensure the final quality of the product [86]. This study monitored the percentage of the viable cells in suspension and the performance of *S. cerevisiae* using 12°P all-malt wort at 15 and 22°C and compared it to fermentations using different concentrations of adjuncts. At 22°C, the concentration of cells in suspension reached a maximum value of 1.0 × 10^8^ cell/mL at 48 h, and at 15°C, the highest number of cells was observed at 60 h (1.8 × 10^8^ cell/mL). After 72 h at 22°C, there was a reduction in the number of viable cells due to the limitation of the substrate and an increase in the concentration of ethanol. At 15°C, this reduction was slower and occurred after 84 h. Many factors can affect the capacity of fermentation by the yeasts; however, the composition of the wort is one of the most important and can vary due to material availability and production techniques [24, 83]. As seen in Figure 4, for all fermentations, there was no adaptative long phase once the substrates were inoculated with new cells and propagated. In the all-malt wort fermentation (0%), the number of viable cells increased and then fell due to flocculation. However, in the fermentation with cocoa pulp as an adjunct under the same conditions at 15°C and 22°C, an increase in the number of cells was observed, characterizing the phase of exponential growth, reaching a maximum point of development and decreasing close to the end of the fermentations. With the use of the adjunct, the growth of cells was faster than in the all-malt wort. Analyzing the results, despite the differentiated performances, the strains of lager and ale *S. cerevisiae* can develop in media of different compositions for beer production and meet the needs of the industry.

The use of an adjunct, in this case, the cocoa pulp, as an alternative method to increase the concentration of soluble solids in the wort favors fermentation and aims at producing a beer with differentiated organoleptic characteristics [24]. The use of certain types of adjuncts as a substitute for part of the malt offers only carbohydrates; therefore, their use in high concentrations can result in the reduction in the content of amino acids available in the wort compared to fermentations without adjuncts [87]. The two nutrients that impact the development of yeasts during fermentation are carbohydrates and nitrogen. The individual assimilation of nutrients depends on the response of the yeasts to the various components. The yeasts *S. cerevisiae* can use several carbohydrates (glucose, sucrose, fructose, maltose, galactose, raffinose, and maltotriose), with differences between the lager and ale strains, as the lager yeasts can ferment melibiose [83].

At 22°C, using the adjunct at the concentrations of 10, 30, and 49%, a higher production of ethanol and a higher concentration of viable cells were observed. As listed in Tables 2 and 3, when analyzing the parameters of fermentation performed at 15 and 22°C, the pulp concentration of 30% shows significant differences (*p* < 0.05) in relation to the other concentrations, with a higher volumetric productivity of ethanol of 0.48 and 0.56 g/L h (Table 7).

A shorter fermentation time occurred when the cocoa pulp was added at the concentration of 30% (Table 8). It is important to note that the experiments performed at 22°C occurred in a shorter time than at 15°C. Briggs et al. [24] found that an increase in the temperature of fermentation causes a reduction in the time of attenuation of the wort because, up to a point, the increase in temperature favors the metabolism of the yeasts studied. The experiments show that the use of 30% cocoa pulp as an adjunct to the malt has the best results with respect to the time of fermentation, apparent degree of fermentation, consumption of substrate, and ethanol production.

According to the results described, the use of an adjunct in a concentration of 30% favored the ethanol production, consumption of substrate, and concentration of cells in suspension in comparison with the all-malt wort at 15°C and 22°C. It was therefore noted that the increase in the concentration of adjunct (up to the limit of 30%) contributed to higher production, indicating a positive effect of the nutrients and sugar profile of the cocoa pulp on the viability and performance of the yeasts during beer fermentation. In this case, the high concentration of fermentable sugars (glucose and fructose) in the cocoa pulp as an adjunct can explain the higher ethanol production. It is important to note that at 49% concentration, the ethanol production was lower than at 30%, which can be related to the reduction of nutrients in the medium due to the high concentration of the adjunct [83–87]. Different concentrations of carbohydrates can negatively affect the fermentation, particularly in worts in which glucose is the predominant carbohydrate [84–88]. In this case, the cocoa pulp shows high concentrations of glucose and fructose at 52.11 and 52.35 g/L, respectively. Therefore, the concentration of 30% cocoa pulp as an adjunct was selected to continue pilot studies at the best performance temperature at 22°C.

## 4. Conclusions

We have studied the application of cocoa pulp as an adjuvant of malt to improve brewing. Based on the results of the characterization of the cocoa pulp, such as pH value of 3.5 and glucose and fructose contents of 52.11 and 52.35 g/L, respectively, the cocoa pulp can be considered to other fruits with potential for use in the beverage industry. The values found for the minerals are also favorable for fermentation, mainly the high contents of potassium, magnesium, and calcium, 1459.842, 237.230, and 13.343 mg/kg, respectively. These minerals can contribute positively to the fermentative performance. The pulp showed an apparent high viscosity of 95 cP at 30°C, which is not appropriate to the fermentation process. Three conditions used to decrease the viscosity of the pulp through variations between the enzyme concentration (polygalacturonase), time, and temperature were able to reduce this characteristic by 65%, which makes the use of any of the conditions for this purpose. The presence of the pulp in the wort in different concentrations (10%, 30%, and 49%) was able to influence its characteristics such as pH (5.30–4.00), glucose (9.87–38.73 g/L), and fructose (1.63–16.43 g/L), for example. Furthermore, the concentration of 30% of the cocoa pulp in the wort was favorable for ethanol production, consumption of substrate, and concentration of cells in suspension in comparison with the all-malt wort at 15°C and 22°C. Thus, the study showed the importance of using the cocoa pulp in the process of obtaining beer.

## Figures and Tables

**Table 1:** Chemical characterization of cocoa pulp, mean values, and standard deviation.

Characteristics	Mean ± standard deviation
Humidity (g/100 g)	86.38 ± 0.09
Ashes (g/100 g)	0.36 ± 0.05
Raw protein (g/100 g)	0.62 ± 0.17
Total lipids (g/100 g)	1.45 ± 0.20
Total sugars (g/100 g)	18.00 ± 0.05
Reducing sugars (g/100 g)	10.41 ± 0.05
Soluble solids (°Brix)	17.00 ± 0.01
Glucose (g/L)	52.11 ± 0.30
Fructose (g/L)	52.35 ± 0.45
pH	3.50 ± 0.01
Total titratable acidity (mEq^−1^ in malic acid)	1.00 ± 0.01
Citric acid (g/L)	8.33 ± 0.60
Malic acid (g/L)	6.10 ± 0.50
Succinic acid (g/L)	0.45 ± 0.34
Lactic acid (g/L)	0.43 ± 0.50
Acetic acid (g/L)	0.40 ± 0.44
Pectin (g/100 g) of calcium pectate	0.51 ± 0.01
Starch (g/100 g)	1.07 ± 0.21
Polyphenol (g/100 g)	0.21 ± 0.02

The values are means of three replicates.

**Table 2:** Determination of the mineral composition of pulp cocoa sample (mg/kg).

Elements	Wavelength *λ* (nm)	LODm (mg/kg)	LOQm (mg/kg)	Mean ± standard deviation
Al	167.019	0.980	3.280	1.742 ± 0.01
Ca	422.673	1348.000	4.490	13.343 ± 0.01
Cd	228.802	0.060	0.196	0.143 ± 0.01
Co	228.615	0.013	0.043	—
Cr	206.158	0.030	0.100	0.970 ± 0.01
Cu	213.598	0.050	0.170	0.419 ± 0.01
Fe	259.940	0.470	1.570	3.388 ± 0.01
K	769.897	0.700	2.338	1459.842 ± 0.01
Li	610.365	0.005	0.017	—
Mg	280.270	0.500	1.670	237.230 ± 0.01
Mn	259.372	0.005	0.015	0.378 ± 0.01
Mo	204.598	0.103	0.340	0.106 ± 0.01
Na	330.298	3.410	11.380	98.966 ± 0.01
P	213.618	0.056	0.185	365.797 ± 0.01
Pb	220.353	0.050	0.168	—
Se	203.985	0.135	0.450	3.251 ± 0.01
V	311.837	0.007	0.022	—
Zn	213.857	0.017	0.058	1.535 ± 0.01

The values are means of three replicates. LOD: limits of detection; LOQ: limits of quantification.

**Table 3:** Coded and uncoded values of the variables studied and percentage of reduction in viscosity in cocoa pulp treated with polygalacturonase from *Aspergillus niger*.

Assay	Enzyme (*μ*L/100 g)	Temperature (°C)	Time (min)	Enzyme (*μ*L/100 g)	Temperature (°C)	Time (min)	Reduction of viscosity (%)
1	−1.000	−1.000	−1.000	282.0	40.0	20.0	50.54
2	−1.000	−1.000	1.000	282.0	40.0	50.0	54.28
3	−1.000	1.000	−1.000	282.0	70.0	20.0	30.86
4	−1.000	1.000	1.000	282.0	70.0	50.0	23.50
5	1.000	−1.000	−1.000	818.0	40.0	20.0	47.18
6	1.000	−1.000	1.000	818.0	40.0	50.0	58.02
7	1.000	1.000	−1.000	818.0	70.0	20.0	42.19
8	1.000	1.000	1.000	818.0	70.0	50.0	26.99
9	−1.682	0.000	0.000	100.0	55.0	35.0	57.14
10	1.682	0.000	0.000	1000.0	55.0	35.0	53.53
11	0.000	−1.682	0.000	550.0	30.0	35.0	53.53
12	0.000	1.682	0.000	550.0	80.0	35.0	0.00
13	0.000	0.000	−1.682	550.0	55.0	10.0	48.67
14	0.000	0.000	1.682	550.0	55.0	60.0	60.76
15	0.000	0.000	0.000	550.0	55.0	35.0	54.65
16	0.000	0.000	0.000	550.0	55.0	35.0	51.04
17	0.000	0.000	0.000	550.0	55.0	35.0	58.40
18	0.000	0.000	0.000	550.0	55.0	35.0	52.66

The values are means of three replicates.

**Table 4:** Experimental conditions and results of the percentage of reduction in the viscosity of the cocoa pulp using pectinolytic enzymes for validation of the model.

Assay	Temperature (°C)	Time (min)	Predicted	Observed	Standard error (%)
1	55.0	47.5	54.72	64.92a	18
2	42.5	60.0	64.37	64.75a	0.59
3	42.5	50.6	77.04	65.71a	14.7

**Table 5:** Chemical analysis of the wort.

Parameters	Pulp percentage (%)			
	0%	10%	30%	49%
pH	5.30^a^ ± 0.01	4.80^b^ ± 0.015	4.50^c^ ± 0.012	4.00^d^ ± 0.02
Humidity (g/100 g)	90.86^a^ ± 0.08	91.02^b^ ± 0.02	91.1^b^ ± 0.021	91.80^c^ ± 0.03
Total titratable acid (meq NaOH/100 g)	1.76^a^ ± 0.05	1.81^b^ ± 0.08	1.85^c^ ± 0.01	1.95^d^ ± 0.04
Reducing sugars (g/100 g)	11.51^a^ ± 0.02	11.64^a^ ± 0.01	17.46^b^ ± 0.01	17.55^b^ ± 0.03
Total sugars (g/100 g)	12.43^a^ ± 0.03	12.22^a^ ± 0.04	17.92^b^ ± 0.06	18.18^b^ ± 0.09
Raw protein (g/100 g)	1.64^a^ ± 0.17	1.60^b^ ± 0.23	1.56^c^ ± 0.25	1.53^d^ ± 0.35
Soluble solids (°Brix)	12.4^a^ ± 0.01	12.5^a^ ± 0.01	14.8^b^ ± 0.01	16.10^c^ ± 0.01
Color (EBC)	15.1^a^ ± 0.02	15.11^a^ ± 0.03	13.8^b^ ± 0.21	12.01^c^ ± 0.15

Values marked with the same identification on the same line between the partial means do not differ significantly (*p* > 0.05), according to the Tukey test.

**Table 6:** Analysis of sugars and organic acids in the wort (g/L).

Parameters	Pulp percentage (%)			
	0%	10%	30%	49%
Glucose	9.87^a^ ± 0.016	16.67^b^ ± 0.02	25.18^c^ ± 0.02	38.73^d^ ± 0.01
Fructose	1.63^a^ ± 0.05	9.45^b^ ± 0.05	11.85^c^ ± 0.03	16.43^d^ ± 0.03
Maltose	58.63^a^ ± 0.02	51.70^b^ ± 0.01	43.85^c^ ± 0.01	26.9^d^ ± 0.01
Maltotriose	14.62^a^ ± 0.03	12.33^b^ ± 0.03	10.77^c^ ± 0.02	9.33^d^ ± 0.01
Citric acid	0.72^a^ ± 0.015	0.86^a^ ± 0.02	2.90^b^ ± 0.04	3.95^c^ ± 0.06
Acetic acid	0.65^a^ ± 0.12	0.64^a^ ± 0.33	0.56^b^ ± 0.35	0.37^c^ ± 0.32
Malic acid	0.2^a^ ± 0.02	0.71^b^ ± 0.015	1.8^c^ ± 0.011	2.5^d^ ± 0.012
Succinic acid	4.44^a^ ± 0.02	3.37^b^ ± 0.02	3.04^c^ ± 0.03	2.13^d^ ± 0.03
Formic acid	0.91^a^ ± 0.02	0.91^a^ ± 0.023	0.93^b^ ± 0.21	0.93^b^ ± 0.15

Values marked with the same identification on the same line between the partial means do not differ significantly (*p* > 0.05), according to the Tukey test.

**Table 7:** Average values with standard deviation of volumetric productivity of ethanol by the commercial yeast for fermentation at 15°C and 22°C with the addition of cocoa pulp at the concentrations of 10%, 30%, and 49% to the malt wort after 96 h of fermentation.

Pulp concentration (%)	Qp (g.L/h)	
	Yeast S-23	Yeast S-04
0	0.43^a^ ± 0.00	0.53^a^ ± 0.00
10	0.47^b^ ± 0.00	0.55^b^ ± 0.00
30	0.48^c^ ± 0.00	0.56^c^ ± 0.00
49	0.47^b^ ± 0.00	0.55^b^ ± 0.00

Values marked with the same identification in the same column between the partial means do not differ significantly (*p* > 0.05), according to the Tukey test.

**Table 8:** Comparison of the time and apparent degree of fermentation (%) for the commercial yeasts S-23 and S-04 in fermentation at 15°C and 22°C, respectively, in all-malt wort using adjunct.

Parameters	Concentration of adjunct							
	0%		10%		30%		49%	
Temperature (°C)	15	22	15	22	15	22	15	22
Time (h)	132	84	84	72	72	60	84	72
Apparent degree of fermentation (%)	74.72^a^ ± 0.1	74.72^e^ ± 0.15	74.92^b^ ± 0.2	74.94^f^ ± 0.18	74.96^c^ ± 0.1	75.01^g^ ± 0.1	74.98^d^ ± 0.2	75.07^h^ ± 0.1

Values marked with the same identification in the same column between the partial means do not differ significantly (*p* > 0.05), according to the Tukey test.

## Data Availability

The data used to support the findings of this study are included within the article.

## Supplementary Materials

Supplementary Materials Table 1S: analysis of variance (ANOVA) for the assay of viscosity reduction in the cocoa pulp. Table 2S: coefficients of regression with all the factors and their interactions for the viscosity reduction analysis assay in the cocoa pulp. Table 3S: coefficients of regression with the factors and significant interactions (*p* < 0.05) for the viscosity reduction analysis assay in the cocoa pulp. Table 4S: *F-*test for the viscosity reduction analysis assay in the cocoa pulp.
