# Supplementary material for: Potential Applicability of Cocoa Pulp (Theobroma cacao L) as an Adjunct for Beer Production
Source: ScientificWorldJournal. 2020 Sep 2;2020:3192585. doi: 10.1155/2020/3192585 (PMC7484685; doi:10.1155/2020/3192585)
Supplement: Supplementary Materials — Table 1S: analysis of variance (ANOVA) for the assay of viscosity reduction in the cocoa pulp. Table 2S: coefficients of regression with all the factors and their interactions for the viscosity reduction analysis assay in the cocoa pulp. Table 3S: coefficients of regression with the factors and significant interactions (p < 0.05) for the viscosity reduction analysis assay in the cocoa pulp. Table 4S: F-test for the viscosity reduction analysis assay in the cocoa pulp. [file 3192585.f1.docx]

**Supplementary Materials**

Four tables of this manuscript represent the additional material.

Below are presented in Tables 1S, 2S, 3S and 4S.

Table 1S refers to Variance analysis (ANOVA) for the assay of viscosity reduction in the cocoa pulp.

Table 2S refers to Coefficients of regression with all the factors and its interactions for the viscosity reduction analysis assay in cocoa pulp.

Table 3S refers to Coefficients of regression with the factors and significant interactions (p<0,05) for the viscosity reduction analysis assay in cocoa pulp.

Table 4S refers to Test F for the viscosity reduction analysis assay in cocoa pulp.

**Table 1S.** Variance analysis (ANOVA) for the assay of viscosity reduction in the cocoa pulp.

| **Parameters** | **Sum of squares** | **Degrees of freedom** | **Average Square** | **Value of F** | **Value of P** |
| --- | --- | --- | --- | --- | --- |
| (1) [ ] enzyme(L) | 6.094 | 1 | 6.094 | 0.607 | 0.493 |
| [ ] enzyme(Q) | 1.352 | 1 | 1.352 | 0.135 | 0.738 |
| (2) Temperature(L) | 2280.760 | 1 | 2280.760 | 227.144 | 0.001 |
| Temperature(Q) | 1375.628 | 1 | 1375.628 | 137.001 | 0.001 |
| (3) Time (L) | 11.195 | 1 | 11.195 | 1.115 | 0.368 |
| Time (Q) | 3.767 | 1 | 3.767 | 0.375 | 0.583 |
| 1L x 2L | 26.107 | 1 | 26.107 | 2.600 | 0.205 |
| 1L x 3L | 0.070 | 1 | 0.070 | 0.007 | 0.939 |
| 2L x 3L | 172.295 | 1 | 172.295 | 17.159 | 0.025 |
| Lack of adjustment | 250.243 | 5 | 50.049 | 4.984 | 0.108 |
| Error | 30.123 | 3 | 10.041 |  |  |
| Total Sum of Squares | 4221.786 | 17 |  |  |  |

R^2^= 93.36%

**Table 2S.** Coefficients of regression with all the factors and its interactions for the viscosity reduction analysis assay in cocoa pulp.

| **Parameters** | **Coefficients of regression** | **Standard Errors** | **T(3)** | **P** | **-95%** | **+95%** |
| --- | --- | --- | --- | --- | --- | --- |
| Interaction | 54,348 | 1,582 | 34,353 | 0,000 | 49,313 | 59.383 |
| 1. [ ] enzyme (L) | 0,668 | 0,857 | 0,779 | 0,493 | -2,061 | 3.397 |
| [ ] enzyme(Q) | -0.323 | 0.891 | -0.367 | 0.738 | -3.162 | 2.508 |
| (2)Temperature(L) | -12.923 | 0.857 | -15.071 | 0.001 | -15.652 | -10.194 |
| Temperature(Q) | -10.428 | 0.891 | -11.705 | 0.001 | -13.264 | -7.593 |
| (3)Time (L) | 0.905 | 0.857 | 1.056 | 0.368 | -1.823 | 3.634 |
| Time (Q) | -0.546 | 0.891 | -0.613 | 0.583 | -3.381 | 2.290 |
| 1L x 2L | 1.806 | 1.120 | 1.613 | 0.205 | -1.759 | 5.372 |
| 1L x 3L | -0.093 | 1.120 | -0.083 | 0.939 | -3.659 | 3.472 |
| 2L x 3L | -4.641 | 1.120 | -4.142 | 0.0256 | -8.206 | -1.075 |

R^2^= 93.36%

**Table 3S.** Coefficients of regression with the factors and significant interactions (p<0,05) for the viscosity reduction analysis assay in cocoa pulp.

| **Parameters** | **Coefficients of regression** | **Standard Errors** | **T(3)** | **P** | **-95%** | **+95%** |
| --- | --- | --- | --- | --- | --- | --- |
| Interaction | 53.571 | 0.991 | 54.070 | 0.000 | 50.418 | 56.724 |
| (2)Temperature(L) | -12.923 | 0.857 | -15.071 | 0.001 | -15.652 | -10.194 |
| (3)Time (L) | -10.277 | 0.858 | -11.978 | 0.001 | -13.008 | -7.547 |
| 2L x 3L | -4.641 | 1.120 | -4.142 | 0.026 | -8.206 | -1.075 |

R^2^= 92.22%

**Table 4S.** Test F for the viscosity reduction analysis assay in cocoa pulp.

| **Source of variation** | **SQ** | **GL** | **QM** | **Fcalc** | **Ftab** |
| --- | --- | --- | --- | --- | --- |
| Regression | 3893.589 | 3 | 1297.863 | 55.36336 | 5.56 (1%) |
| Residue | 328.197 | 14 | 23.443 |  | 3.34 (5%) |
| Lack of Adjustment | 298.074 | 11 |  |  | 2.2 (10%) |
| Pure error | 30.123 | 3 |  |  |  |
| Total | 4221.786 | 17 |  |  |  |
